# Effect of Graded-Indium-Content Superlattice on the Optical and Structural Properties of Yellow-Emitting InGaN/GaN Quantum Wells

**DOI:** 10.3390/ma14081877

**Published:** 2021-04-09

**Authors:** Xuan Li, Jianping Liu, Xujun Su, Siyi Huang, Aiqin Tian, Wei Zhou, Lingrong Jiang, Masao Ikeda, Hui Yang

**Affiliations:** 1 Suzhou Institute of Nano-Tech and Nano-Bionics, Chinese Academy of Sciences, Suzhou 215123, China; xli2019@sinano.ac.cn (X.L.); syhuang2015@sinano.ac.cn (S.H.); aqtian2012@sinano.ac.cn (A.T.); wzhou2015@sinano.ac.cn (W.Z.); lrjiang2016@sinano.ac.cn (L.J.); mikeda2013@sinano.ac.cn (M.I.); hyang2006@sinano.ac.cn (H.Y.); 2 Key Laboratory of Nanodevices and Applications, Suzhou Institute of Nano-Tech and Nano-Bionics, Chinese Academy of Sciences, Suzhou 215123, China; 3 Nano Science and Technology Institute, University of Science and Technology of China, Suzhou 215123, China; 4 School of Nano-Tech and Nano-Bionics, University of Science and Technology of China, Hefei 230026, China

**Keywords:** GaN, superlattice, InGaN, multiple quantum wells

## Abstract

We have improved the material quality of the high indium composition InGaN/GaN multiple quantum wells (MQWs) grown on free-standing GaN substrates using the graded-indium-content superlattice. We found that by adopting a graded-indium-content superlattice structure, the spectral FWHM of the yellow emitting InGaN/GaN MQW was reduced from 181 meV to 160 meV, and the non-radiative recombination lifetime increased from 13 ns to 44 ns. Besides, the graded-indium-content superlattice can mitigate strain relaxation in high indium composition MQWs as shown by the TEM diffraction patterns.

## 1. Introduction

GaN-based emitters such as light-emitting diodes (LED) and laser diodes (LD) have made great success in the past decades, especially in the blue spectrum range [1,2,3,4,5]. However, when the wavelength extends to a longer range, corresponding to the maximum sensitivity of human eyes, the emitting efficiency of multi-quantum wells (MQWs) drops sharply which is known as the “green gap” [6,7,8]. The reason for the green gap can be attributed to the increasing non-radiative recombination rates caused by deteriorative material quality [9,10], a more severe Quantum Confinement Stark Effect (QCSE) [11,12], and the Auger effect. To overcome the gap, considerable approaches have been proposed by researchers. For example, nonpolar or semipolar substrates were used to control QCSE [13,14,15,16]. Blue MQWs were introduced as strain modulation layers to improve material quality and reduce strain in yellow MQWs as reported by Zhang et al. [17]. AlGaN interlayer (IL) was used as band engineering and strain compensation of active layers to enhance IQE [18,19,20,21]. Some literature reported the application of an InGaN underlayer to reduce potential point defects in the underlayer, and therefore, boost MQWs efficiency [22,23,24]. Specifically, Jiang’s team [25,26,27,28] came up with a pre-strained superlattice (SL) structure to solve the phase separation problem and improve the crystalline quality in high indium MQWs, assisting in realizing high performances light-emitting diodes in longer wavelength. However, for laser diodes, GaN free-standing substrates rather than foreign substrates are applied to decrease threading dislocations and improve material quality. There are few studies about the influence of pre-strained SL on homoepitaxial long-wavelength MQWs performances. The effect of the pre-strained superlattice structure on the homoepitaxial growth remains to be studied.

## 2. Experimental

**Figure 1:**
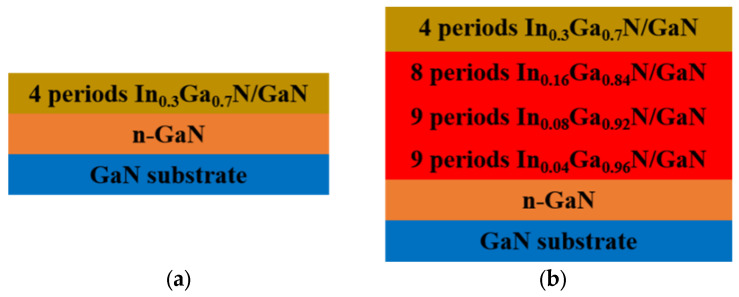
Cross-sectional schematic diagram of quantum well’s structure: (**a**) 4 MQWs without GSLs (**b**) 4 MQWs with GSLs.

In this experiment, the epitaxial growth of the samples was performed on a commercial Aixtron 6 × 2 inch Closed Coupled Shower Head (CCS) metal-organic chemical vapor deposition (MOCVD) reactor. Trimethylindium (TMIn) and ammonia (NH_3_) were used as precursors for In and N, respectively. Trimethylgallium (TMGa) was used as a precursor for Ga when growing the GaN layer while triethylgallium (TEGa) was used as a precursor for Ga when growing the InGaN layer. Nitrogen and hydrogen were used as carrier gases. Monosilane (SiH_4_) was used as n-type dopants. Sample A and B were grown on c-plane free-standing GaN substrates as shown in Figure 1a,b, respectively. Sample A was a traditional InGaN/GaN MQWs and sample B was an InGaN/GaN MQW with a graded-indium-content InGaN/GaN superlattice (GSLs) as the pre-strained structure. The growth conditions of MQWs of sample A and sample B were completely the same. The growth pressure was 400 mbar. The specific growth parameters are shown in Table 1. The growth temperature was thermocouple temperatures. The thickness of QWs and QBs were 2.5 nm and 17 nm, and the thickness of InGaN and GaN of SGLs were 5.5 nm and 1.5 nm, respectively.

Room-temperature photoluminescence (PL) measurement was performed with a 325 nm semiconductor laser diode with an excitation density of 1.6 W cm^−2^. A digital modulated (10 kHz, 50% duty cycle) 405 nm CW semiconductor laser diode was used as the excitation source in the time-resolved photoluminescence (TRPL) experiment and the injected power density was kept around 5.7 W cm^−2^. Transmission electron microscopy (TEM), scanning electron microscope (SEM), and atomic force microscopy (AFM) were used to observe and analyze dislocations in MQWs. AFM was the Dimension ICON produced by Bruker in the United States. SEM was S-4800 produced by HITACHI in Japan. TEM was Talos F200X and Themis Z produced by FEI in the United States.

## 3. Results and Discussion

**Figure 2:**
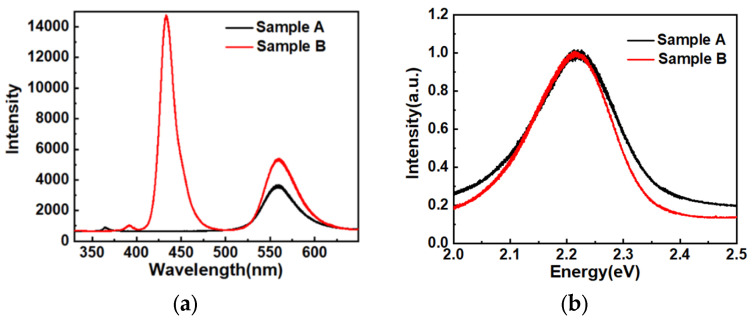
(**a**) PL spectra of samples A and B recorded at room temperature; (**b**) Normalized spectra of samples A and B with the energy-scale horizontal axis.

The room temperature PL spectra of samples A and B are shown in Figure 2. The PL peak energy and the full width at half maximum (FWHM) of each spectrum were determined by peak fitting. The black and the blue curves represent the room temperature PL spectrum of samples A and B, respectively. It could be found that the peak emission energy of sample A and sample B was the same, 2.214 eV. On the other hand, the FWHM of PL peak of sample A and sample B was 181 meV and 160 meV, respectively.

To figure out the reason for improved material quality, time resolve photoluminescence (TRPL) measurements were conducted. We used a novel method to determine both the radiative and non-radiation lifetime of excess carriers at room temperature. The related theoretical explanation could be found in References [29,30]. A modulated quasi-CW laser was used as the excitation source in the TRPL experiment. From the moment when the excitation was turned off until the photoluminescence intensity dropped to 1/e of the steady-state intensity, we used the formula $I_{PL,deacy}\;\propto e^{-t/\tau_{eff}}$ to fit the curve of the decay of the photoluminescence intensity with time to a single exponential function as the effective lifetime of the carrier under steady-state excitation. The $\tau_{eff}$ is the effective lifetime of excess carriers and is given by:

(1) $$\frac{1}{\tau_{eff}}=\frac{1}{\tau_{rad}}+\frac{1}{\tau_{SRH}}+\frac{1}{\tau_{Auger}}$$

where *τ_rad_*, *τ_SRH_* and *τ_Auger_* are the lifetimes of radiative, SRH, and Auger recombination, respectively. Under low excitation, the SRH lifetime is the main factor affecting the effective lifetime because the radiative recombination and Auger recombination process are slow. Therefore, it can be considered that non-radiative recombination is equivalent to SRH recombination.

**Figure 3:**
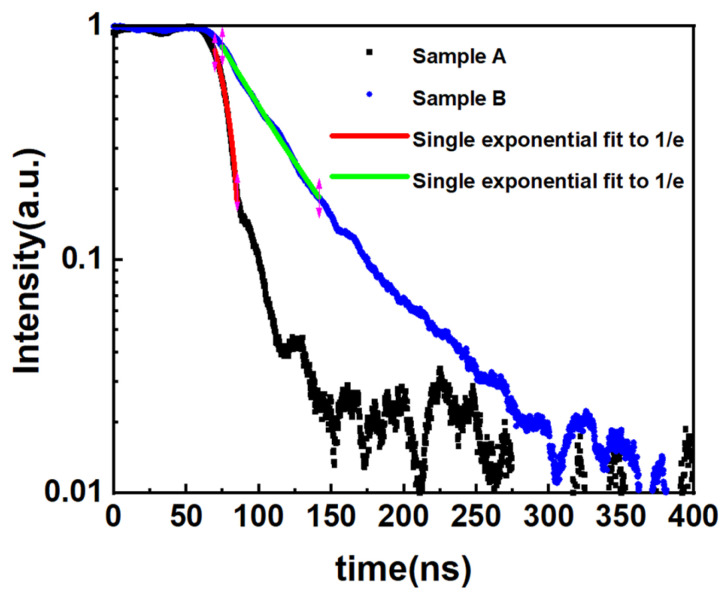
Normalized PL intensity decay curves of samples A and B recorded at room temperature.

The curve of TRPL at room temperature in this experiment is shown in Figure 3. The black and the red curves represent PL decay curves of samples A and B, respectively. The non-radiative recombination lifetime of sample A and sample B was 13 ns and 44 ns, respectively. The lifetime of non-radiative recombination was closely related to the density defects. It was reasonable to believe sample B had fewer non-radiative recombination centers, and therefore, had a better material quality. This result agreed well with the PL measurement results. The reason was that native defects such as V_N_ were trapped by the indium atom in the pre-strained well to form the In-V_N_ with lower energy [31,32,33]; therefore, reducing the number of defects in the subsequently grown quantum well layer. Thus, the FWHM of the PL spectrum was reduced and the non-radiative recombination lifetime was increased.

**Figure 4:**
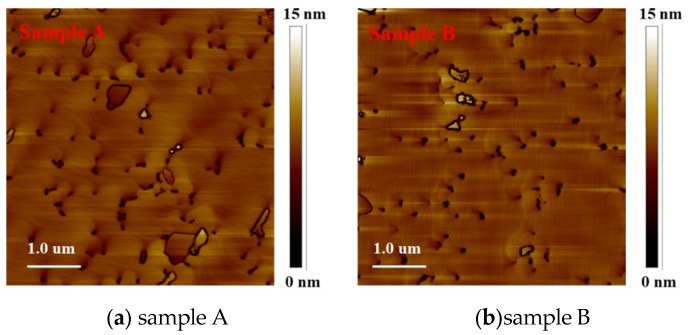
AFM images of yellow MQWs on freestanding GaN substrate (**a**) sample A and (**b**) sample B.

**Figure 5:**
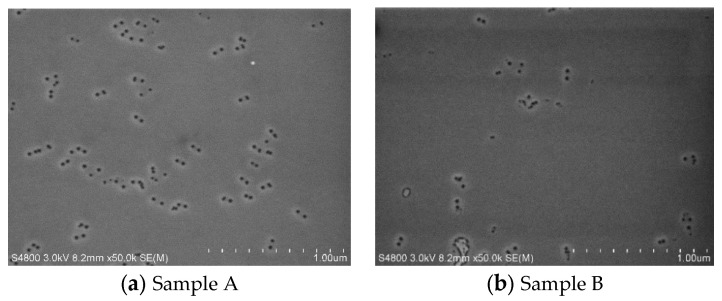
SEM images of yellow MQWs on freestanding GaN substrate of MQWs In (**a**) sample A and (**b**) sample B.

AFM and SEM images are shown in Figure 4 and Figure 5, respectively. The scan size of AFM images was 5 um × 5 um. We can see through the AFM and SEM images that there were many V-pits on the surface of two samples and most of the V-pits appear in pairs. The dislocations density of free-standing GaN substrates was less than 3 × 10^6^/cm^2^. However, it can be seen that the dislocations density of sample A and sample B on the surface are 10^7^/cm^2^ through the AFM images. We think the reason was a large lattice mismatch between the InGaN and GaN in the high indium composition MQWs, which results in misfit dislocations.

**Figure 6:**
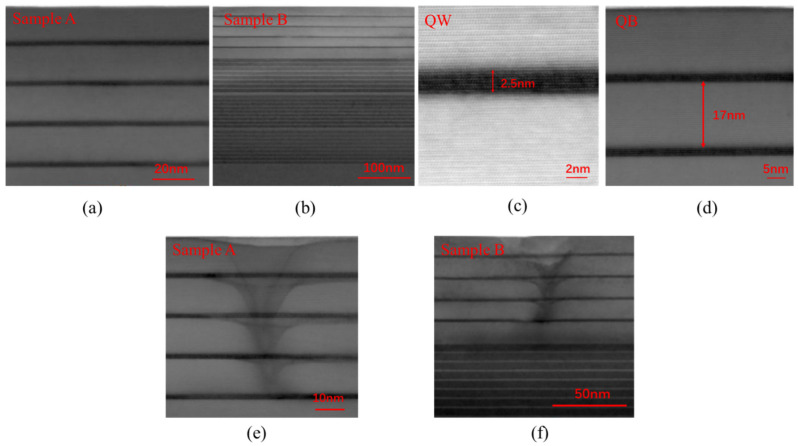
Cross-sectional bright-field STEM images of yellow MQWs on freestanding GaN substrate (**a**) sample A, and (**b**) sample B, (**c**) the thickness of QW, (**d**) the thickness of QB, (**e**) a dislocation in sample A (**f**) a dislocation in sample B.

**Figure 7:**
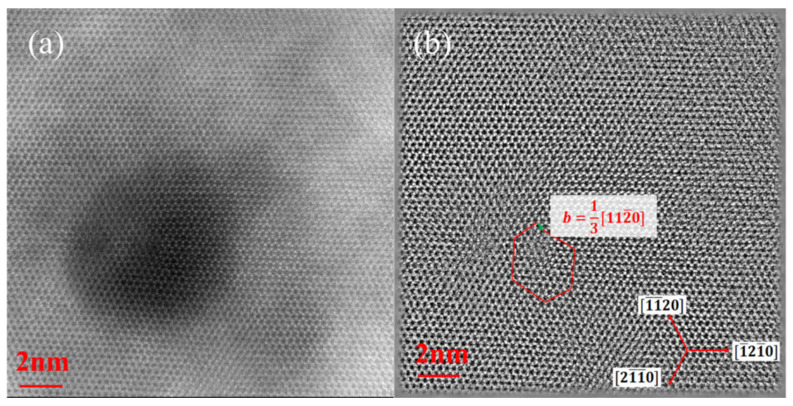
(**a**) Plan-view atomic resolution high-angle annular dark-field STEM images of a V-pit. (**b**) was obtained by inverse Fourier transform after filtering out the (0000) from the diffraction pattern obtained by Fourier transformation of (**a**).

To verify this conclusion, the formation of dislocations and the strain in MQWs was studied. Figure 6 shows bright-field scanning transmission electron microscope (STEM) images of samples A and B. As shown in Figure 6a,b, cross-sectional STEM of sample A and sample B show that the interface between the high indium composition yellow-emitting InGaN QWs and the GaN QBs is clear. Figure 6c,d show that the thickness of QWs and QBs is 2.5 nm and 17 nm, respectively. Dislocations also appeared from the first or second QW layer in both samples from the cross-sectional STEM as shown in Figure 6e,f. Figure 7a is a plan-view atomic resolution high-angle annular dark-field (HAADF) STEM image of a V-pit. The dark contrast was caused by the smaller thickness within the V-pit. Figure 7b was obtained by inverse Fourier transform after filtering out the (0000) from the reciprocal lattice obtained by Fourier transformation of Figure 7a. It showed a dislocation was formed within the red lines with Burgers vector of $\frac{1}{3}\left[11\bar{2}0\right]$.

**Figure 8:**
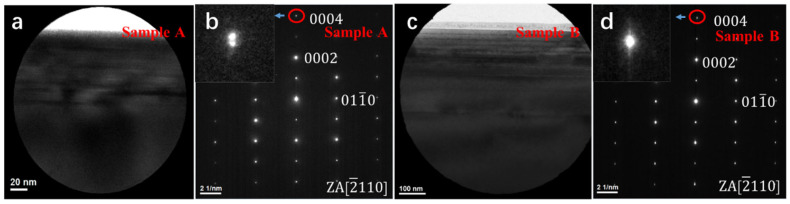
(**a**) Cross-sectional TEM image of sample A; (**b**) Selected area electron diffraction pattern of Figure 8a taken under zone-axis $\left[\bar{2}110\right]$; (**c**) Cross-sectional TEM image of sample B; (**d**) Selected area electron diffraction pattern of Figure 8a taken under zone-axis $\left[\bar{2}110\right]$.

Next, we used selected area electron diffraction (SAED) to characterize the strain in the MQWs. Figure 8a is a cross-sectional view image of sample A. Figure 8b is a SAED pattern of the circled area of Figure 8a taken under zone axis (ZA) $\left[\bar{2}110\right]$. Figure 8c is a cross-sectional view image of sample B. Figure 8d is a diffraction pattern of the circled area of Figure 8c also under ZA $\left[\bar{2}110\right]$. The circled areas in both Figure 8a,c contain MQWs and n-GaN layer. The inset images of Figure 8b,d are enlarged views of the diffraction spots of 0004 of sample A and sample B, respectively. In Figure 8b, we can see that the diffraction spots of 0004 of sample A are two spots, which means that the lattice constants of InGaN and GaN are different. It shows that relaxation has occurred in sample A. In Figure 8d, the diffraction spots of 0004 of sample B are a slightly elongated dot. Combined with the AFM image of sample B, it can be seen that relaxation also occurred in sample B, but the resolution of the diffraction pattern was not enough to distinguish them. According to the comparison of the diffraction patterns of 0004 of the two samples, it could be concluded that the relaxation of sample B was smaller than that of sample A. Therefore, the GSLs can play a key role to mitigate the strain in InGaN QWs.

## 4. Conclusions

We studied the effect of the graded-indium-content superlattice structure on the optical and structural properties of high indium composition yellow-emitting InGaN/GaN MQWs grown on free-standing GaN substrates. It is found that by adopting a graded-indium-content superlattice structure, the spectral FWHM of the yellow emitting InGaN/GaN MQW is reduced from 181 meV to 160 meV, and the non-radiative recombination lifetime is increased from 13 ns to 44 ns. The TEM diffraction patterns prove that the GSLs can mitigate strain relaxation in high indium composition MQWs.

## Figures and Tables

**Table 1:** Growth parameters of different samples.

Scheme	T_set_(°C)	Growth Rate (nm/s)
QW	830	0.033
QB	1005	0.063
GSL (16% InGaN)	903	0.022
GSL (8% InGaN)	903	0.02
GSL (4% InGaN)	903	0.018
GSL (GaN)	903	0.017

## Data Availability

The data presented in this study are available on request from the corresponding author.

## References

[1] Nakamura S., Senoh M., Nagahama S.-I., Iwasa N., Matsushita T., Mukai T. (2000). Blue InGaN-based laser diodes with an emission wavelength of 450 nm. Appl. Phys. Lett..

[2] Zhong Z., Lu S., Li J., Lin W., Huang K., Li S., Cai D., Kang J. (2021). Design and fabrication of high power InGaN blue laser diode over 8 W. Opt. Laser Technol..

[3] Nakamura S., Mukai T., Senoh M. (1994). Candela-class high-brightness InGaN/AlGaN double-heterostructure blue-light-emitting diodes. Appl. Phys. Lett..

[4] Liu J.P., Zhang L.Q., Li D.Y., Zhou K., Cheng Y., Zhou W., Tian A.Q., Ikeda M.S., Zhang S.M., Yang H. (2017). GaN-Based Blue Laser Diodes With 2.2 W of Light Output Power Under Continuous-Wave Operation. IEEE Photonics Technol. Lett..

[5] Narukawa Y., Ichikawa M., Sanga D., Sano M., Mukai T. (2010). White light emitting diodes with super-high luminous efficacy. J. Phys. D Appl. Phys..

[6] Nakamura S., Senoh N., Iwasa N., Nagahama S.I. (1995). High-brightness InGaN blue, green and yellow light-emitting-diodes eith quantum-well structures. Jpn. J. Appl. Phys. Part 2 Lett. Express Lett..

[7] O’Donnell K.P., Auf der Maur M., Di Carlo A., Lorenz K., The Sorbet Consortium (2012). It’s not easy being green: Strategies for all-nitrides, all-colour solid state lighting. Phys. Status Solidi RRL.

[8] Krames M.R., Shchekin O.B., Mueller-Mach R., Mueller G.O., Zhou L., Harbers G., Craford M.G. (2007). Status and future of high-power light-emitting diodes for solid-state lighting. J. Disp. Technol..

[9] Langer T., Kruse A., Ketzer F.A., Schwiegel A., Hoffmann L., Jönen H., Bremers H., Rossow U., Hangleiter A. (2011). Origin of the “green gap”: Increasing nonradiative recombination in indium-rich GaInN/GaN quantum well structures. Phys. Status Solidi C.

[10] Avramescu A., Lermer T., Müller J., Eichler C., Bruederl G., Sabathil M., Lutgen S., Strauss U. (2010). True Green Laser Diodes at 524 nm with 50 mW Continuous Wave Output Power onc-Plane GaN. Appl. Phys. Express.

[11] Chichibu S., Azuhata T., Sota T., Nakamura S. (1996). Spontaneous emission of localized excitons in InGaN single and multiquantum well structures. Appl. Phys. Lett..

[12] Bernardini F., Fiorentini V., Vanderbilt D. (1997). Spontaneous polarization and piezoelectric constants of III-V nitrides. Phys. Rev. B.

[13] Enya Y., Yoshizumi Y., Kyono T., Akita K., Ueno M., Adachi M., Sumitomo T., Tokuyama S., Ikegami T., Katayama K. (2009). 531 nm Green Lasing of InGaN Based Laser Diodes on Semi-Polar {20\bar21} Free-Standing GaN Substrates. Appl. Phys. Express.

[14] Yoshizumi Y., Adachi M., Enya Y., Kyono T., Tokuyama S., Sumitomo T., Akita K., Ikegami T., Ueno M., Katayama K. (2009). Continuous-Wave Operation of 520 nm Green InGaN-Based Laser Diodes on Semi-Polar {20\bar21} GaN Substrates. Appl. Phys. Express.

[15] Takagi S., Enya Y., Kyono T., Adachi M., Yoshizumi Y., Sumitomo T., Yamanaka Y., Kumano T., Tokuyama S., Sumiyoshi K. (2012). High-Power (over 100 mW) Green Laser Diodes on Semipolar {20-21} GaN Substrates Operating at Wavelengths beyond 530 nm. Appl. Phys. Express.

[16] Sizov D., Bhat R., Heberle A., Visovsky N., Zah C.-E. (2011). True-green (11-22) plane optically pumped laser with cleaved m-plane facets. Appl. Phys. Lett..

[17] Zhang J., Xiong C., Liu J., Quan Z., Wang L., Jiang F. (2014). High brightness InGaN-based yellow light-emitting diodes with strain modulation layers grown on Si substrate. Appl. Phys. A.

[18] Saito S., Hashimoto R., Hwang J., Nunoue S. (2013). InGaN Light-Emitting Diodes onc-Face Sapphire Substrates in Green Gap Spectral Range. Appl. Phys. Express.

[19] Shioda T., Yoshida H., Tachibana K., Sugiyama N., Nunoue S. (2012). Enhanced light output power of green LEDs employing AlGaN interlayer in InGaN/GaN MQW structure on sapphire (0001) substrate. Phys. Status Solidi A.

[20] Koleske D.D., Fischer A.J., Bryant B.N., Kotula P.G., Wierer J.J. (2015). On the increased efficiency in InGaN-based multiple quantum wells emitting at 530–590 nm with AlGaN interlayers. J. Cryst. Growth.

[21] Al Muyeed S.A., Sun W., Wei X., Song R., Koleske D.D., Tansu N., Wierer J.J. (2017). Strain compensation in InGaN-based multiple quantum wells using AlGaN interlayers. Aip Adv..

[22] Akasaka T., Gotoh H., Saito T., Makimoto T. (2004). High luminescent efficiency of InGaN multiple quantum wells grown on InGaN underlying layers. Appl. Phys. Lett..

[23] Armstrong A.M., Bryant B.N., Crawford M.H., Koleske D.D., Lee S.R., Wierer J.J. (2015). Defect-reduction mechanism for improving radiative efficiency in InGaN/GaN light-emitting diodes using InGaN underlayers. J. Appl. Phys..

[24] Haller C., Carlin J.F., Jacopin G., Martin D., Butté R., Grandjean N. (2017). Burying non-radiative defects in InGaN underlayer to increase InGaN/GaN quantum well efficiency. Appl. Phys. Lett..

[25] Jiang F., Zhang J., Xu L., Ding J., Wang G., Wu X., Wang X., Mo C., Quan Z., Guo X. (2019). Efficient InGaN-based yellow-light-emitting diodes. Photonics Res..

[26] Jiang X.G., Zheng C.D., Mo C.L., Wang X.L., Zhang J.L., Quan Z.J., Liu J.L., Jiang F.Y. (2019). Study on the performance of InGaN-based green LED by designing different preparing layers. Opt. Mater..

[27] Qi W.J., Zhang J.L., Mo C.L., Wang X.L., Wu X.M., Quan Z.J., Wang G.X., Pan S., Fang F., Liu J.L. (2017). Effects of thickness ratio of InGaN to GaN in superlattice strain relief layer on the optoelectrical properties of InGaN-based green LEDs grown on Si substrates. J. Appl. Phys..

[28] Tao X., Liu J., Zhang J., Mo C., Xu L., Ding J., Wang G., Wang X., Wu X., Quan Z. (2018). Performance enhancement of yellow InGaN-based multiple-quantum-well light-emitting diodes grown on Si substrates by optimizing the InGaN/GaN superlattice interlayer. Opt. Mater. Express.

[29] Zhou R., Ikeda M., Zhang F., Liu J., Zhang S., Tian A., Wen P., Li D., Zhang L., Yang H. (2019). Steady-state recombination lifetimes in polar InGaN/GaN quantum wells by time-resolved photoluminescence. Jpn. J. Appl. Phys..

[30] Zhou R., Ikeda M., Zhang F., Liu J., Zhang S., Tian A., Wen P., Li D., Zhang L., Yang H. (2020). Total-InGaN-thickness dependent Shockley-Read-Hall recombination lifetime in InGaN quantum wells. J. Appl. Phys..

[31] Haller C., Carlin J.F., Jacopin G., Liu W., Martin D., Butté R., Grandjean N. (2018). GaN surface as the source of non-radiative defects in InGaN/GaN quantum wells. Appl. Phys. Lett..

[32] Armstrong A., Henry T.A., Koleske D.D., Crawford M.H., Lee S.R. (2012). Quantitative and depth-resolved deep level defect distributions in InGaN/GaN light emitting diodes. Opt. Express.

[33] Haller C., Carlin J.-F., Mosca M., Rossell M.D., Erni R., Grandjean N. (2019). InAlN underlayer for near ultraviolet InGaN based light emitting diodes. Appl. Phys. Express.

